# A Rare HBV Subgenotype D4 with Unique Genomic Signatures Identified in North-Eastern India –An Emerging Clinical Challenge?

**DOI:** 10.1371/journal.pone.0109425

**Published:** 2014-10-08

**Authors:** Priyanka Banerjee, Rajiv Kumar Mondal, Madhuparna Nandi, Sumantra Ghosh, Mousumi Khatun, Nabendu Chakraborty, Swatilekha Bhattacharya, Arindam RoyChoudhury, Soma Banerjee, Amal Santra, Samir Sil, Abhijit Chowdhury, Pradip Bhaumik, Simanti Datta

**Affiliations:** 1 Centre for Liver Research, School of Digestive and Liver Diseases, Institute of Post Graduate Medical Education and Research, Kolkata, India; 2 Agartala Government Medical College, Tripura, India; 3 Columbia University, New York, New York, United States of America; 4 Tripura University, Tripura, India; 5 Department of Hepatology, School of Digestive and Liver Diseases, Institute of Post Graduate Medical Education and Research, Kolkata, India; Saint Louis University, United States of America

## Abstract

**Background/Aims:**

HBV has been classified into ten genotypes (A–J) and multiple subgenotypes, some of which strongly influence disease outcome and their distribution also correlate with human migration. HBV infection is highly prevalent in India and its diverse population provides an excellent opportunity to study the distinctiveness of HBV, its evolution and disease biology in variegated ethnic groups. The North-East India, having international frontiers on three sides, is one of the most ethnically and linguistically diverse region of the country. Given the paucity of information on molecular epidemiology of HBV in this region, the study aimed to carry out an in-depth genetic characterization of HBV prevailing in North-East state of Tripura.

**Methods:**

From sera of chronically HBV infected patients biochemical/serological tests, HBV DNA quantification, PCR-amplification, sequencing of PreS/S or full-length HBV genomes were done. HBV genotype/subgenotype determination and sequence variability were assessed by MEGA5-software. The evolutionary divergence times of different HBV subgenotypes were estimated by DNAMLK/PHYLIP program while jpHMM method was used to detect any recombination event in HBV genomes.

**Results:**

HBV genotypes D (89.5%), C (6.6%) and A (3.9%) were detected among chronic carriers. While all HBV/A and HBV/C isolates belonged to subgenotype-A1 and C1 respectively, five subgenotypes of HBV/D (D1–D5) were identified including the first detection of rare D4. These non-recombinant Indian D4 (IndD4) formed a distinct phylogenetic clade, had 2.7% nucleotide divergence and recent evolutionary radiation than other global D4. Ten unique amino acids and 9 novel nucleotide substitutions were identified as IndD4 signatures. All IndD4 carried T^120^ and R^129^ in ORF-S that may cause immune/vaccine/diagnostic escape and N^128^ in ORF-P, implicated as compensatory Lamivudine resistance mutation.

**Conclusions:**

IndD4 has potential to undermine vaccination programs or anti-viral therapy and its introduction to North-East India is believed to be linked with the settlement of ancient Tibeto-Burman migrants from East-Asia.

## Introduction

The striking genetic heterogeneity displayed by HBV result mostly from copy errors introduced by viral polymerase during replication of its DNA genome through reverse transcription of pregenomic RNA [1]. However, the long-term fate of these abundant genetic changes depend upon the interactions of HBV with its host, the dramatic intra-host diversification and on different genetic processes such as selection, recombination, genetic drift, population dynamics, and biogeography [1]. Comparisons of HBV sequences from different geographical regions revealed the presence of ten genotypes (A–J), defined by genome dissimilarity by more than 7.5% and most genotypes further segregate into subgenotypes that differ from each other by 4–7.5% [1]. Among them, Genotype D (HBV/D) is most widespread and comprised of 9 subgenotypes (D1–D9), of which D1–D3 appear worldwide while D4–D9 have a more restricted distribution [1], [2].

HBV infection is extremely common in India that is remarkable for its rich population genomic diversity [3]. Taking into consideration the parallelism between HBV-host co-evolution, the diverse Indian population provides an excellent opportunity to study the underpinnings of HBV diversity and disease biology. Of the three genotypes of HBV, D, A and C found in India, HBV/D is predominant and five D-subgenotypes, D1, D2, D3, D5 and D9 had been reported with different local distributions [1], [3]. However, there is very limited information on molecular epidemiology of HBV from North-East (N-E) India that has always been a hotspot for population geneticists due to the presence of linguistically, culturally and demographically varied population. Owing to its strategic geographic location, being hemmed in by countries like China, Myanmar, Bhutan and Bangladesh on three sides, the N-E region serve as a gateway connecting India to East/South-East Asia and had been a corridor of extensive human migration in the past. Moreover, studies on N-E Indian populations indicated that their mitochondrial DNA and Y-chromosome gene pools show closer genetic affinities with East/South-East Asian groups than with mainland Indian population [4].

Seven contiguous States (Arunachal Pradesh, Assam, Manipur, Meghalaya, Mizoram, Nagaland and Tripura) make up the N-E part of the country of which Tripura is the smallest state with a population of about 36.71 lakhs (2011 census). It had a predominantly tribal population in the past but the huge wave of immigration from neighboring Bangladesh in post-independence era had resulted in its demographic transformation rendering its indigenous tribal population to a minority [5]. Hepatitis B positivity has been found to be about 4% in Tripura [6]. We initiated the present study to assess the distribution of viral genotypes/subgenotypes in chronically HBV infected patients of Tripura and in the process report for the first time the presence of subgenotype D4 in India and the emergence unique molecular signatures in these isolates that distinguished them from previously reported D4 strains and results in their separate clustering as a new clade that we designate as Indian D4 (IndD4).

## Materials and Methods

### Collection of blood samples

Five millilitre blood was collected with informed written consent from seventy six treatment-naïve chronically HBV infected patients reporting at Agartala Government Medical College, Agartala, Tripura, between 2010 to 2012. Patients with other viral infections and history of significant alcohol intake were excluded. The serum was separated and stored at −80°C until use. The study was approved by Ethical Review Committee of Agartala Government Medical College, Agartala, Tripura.

### Testing of serological markers and liver enzymes

Each serum sample was tested for the levels of liver enzymes alanine aminotransferase (ALT) and aspartate aminotransferase (AST) using commercially available kits from Bayer Diagnostics (India) in a semiautomated biochemistry analyzer (RA-50; Bayer). The normal ranges for AST and ALT are 2 to 45 IU/liter and 2 to 40 IU/liter, respectively. Serological markers for hepatitis B virus surface antigen (HBsAg) and hepatitis B virus e antigen (HBeAg) and antibodies to HBeAg (anti-HBeAg) were also checked in each sample, using commercially available enzyme-linked immunosorbent assay (ELISA) kits from General Biologicals, Taiwan, and bioMe'rieux, Boxtel, Netherlands.

### HBV DNA isolation and quantification

HBV DNA was extracted from 200 µl sera using QIAamp DNA Mini kit (Qiagen Inc., Valencia, CA). Viral DNA titer was determined by real time PCR using Roche Diagnostics Cobas Taqman 48 (Roche Diagnostics Molecular Systems, Alameda, CA) and the lower limit of detection was 20 IU/ml.

### Amplification and sequencing of HBV genome

The preS1/preS2/S region of HBV was amplified from each sample by PCR using primers F8 and R9 (Table 1) while for samples with low viral load, a second round of amplification with nested primer pair F3 and R8 was performed. Similarly, amplification of basal core promoter (BCP) and Precore (PC) region of HBV was carried out with primer pairs F7-R2 and F1-SP2 (Table 1) in single or two-step nested PCR reactions as appropriate. From selected samples full length HBV DNA was amplified with primers P1 and P2 (Table 1). In case of low HBV DNA level, a second round nested PCR was subsequently performed using two different primer sets, MP1 with R5 and F3 with MP2 [1]. All PCR products were purified by the QIA quick Gel Extraction Kit (Qiagen, CA) and the nucleotide sequences were determined using BigDye terminator cycle sequencing kit (AB Applied Biosystems, Foster City, CA) on automated DNA sequencer (ABI Prism 3130). DNA sequence editing and analysis were performed using Seqscape V2.5 (Applied Biosystems) software.

**Table 1:** Primers used in this study.

Primer Name	Primer sequence	Location [nt**]
F1	5′-CACAAGAGGACTCTTGGACT-3′	1653–1672
F3	5′-CGCCTCATTTTGTGGGTCAC-3′	2801–2820
F4	5′-CTCAGGCCATGCAGTGGAA-3′	3164–3182
F5	5′-GATGTGTCTGCGGCGTTTTA-3′	376–395
F7	5′ -TGTGCACTTCGCTTCACCTC -3′	1578–1597
F8	5′ -ATTTGCATACTCTTTGGAAGGC-3′	2746–2767
F10	5′ -GACCACCAAATGCCCCTATC -3′	2298–2317
R1	5′-CCACCTTATGAGTCCAAGG-3′	2457–2475
R2	5′-AAATTACCACCCACCCAGG-3′	2109–2127
R3	5′-AACTGGAGCCACCAGCAG-3′	57–74
R5	5′-AAAGCCCAAAAGACCCACAAT-3′	996–1016
R8	5'-CTTTGACAAACTTTCCAATCAAT- 3'	973–995
R9	5'-TAGGAGTTCCGCAGTATGGA- 3	1265–1284
SP2	5′-GTATGGTGAGGTGAACAATG-3′	2039–2058
MP1	5′-GAGCTCTTCTTTTTCACCTCTGCCTAATCA-3′	1821–1841
MP2	5′-GAGCTCTTCAAAAAGTTGCATGGTGCTGG-3′	1806–1825
P1ψ	5' -CCGGAAAGCTTGAGCTCTTCTTTTTCACCTCTGCCTAATCA- 3′	1821–1841
P2ψ	5'-CGGAAAGCTTGAGCTCTTCAAAAAGTTGCATGGTGCTGG- 3′	1823–1806

**  =  nucleotide [nt] positions are given according to HBV sequence with accession no. AF121242 obtained from GenBank.

ψ Gunther S *et al*. J. Virol. 1995; 69: 5437–44.

### Phylogenetic analyses of HBV sequences and estimation of divergence time

To determine HBV genotype/subgenotype, preS/S as well as full-length HBV sequences obtained in the study were compared with representative sequences of 10 genotypes (A–J) as well as that of subgenotypes of A (A1–A7), C (C1–C16) and D (D1–D9) retrieved from GenBank. Sequence alignments were carried out using CLUSTAL_X software and phylogenetic trees were constructed by neighbour-joining method using Kimura 2 parameter model in MEGA software version-5 (www.megasoftware.net). To confirm the reliability of phylogenetic tree analysis, bootstrap resampling and reconstruction were carried out 5000 times. Sequence variability was analyzed with the help of multiple alignment data. The evolutionary relationship between the non-recombinant D-subgenotypes (D1 to D7) was examined using a likelihood method of the DNAMLK program of PHYLIP package (http://evolution.genetics.washington.edu/phylip.html), which uses a ‘molecular clock’ assumption.

### Investigation of Recombination events

To detect recombination events in HBV genome sequences, a circular version of jumping profile hidden Markov model (jpHMM) available online at http://jphmm.gobics.de/submission_hbv.htm was used as described previously [1].

### Statistical analysis

The data were analyzed in GraphPad PRISM v5 software. The results were presented as median (range) or mean± standard deviation (SD) as applicable. For comparison of quantitative variable, one-way ANOVA or the Mann-Whitney tests were used and for all tests performed, a p-value <0.05 was considered as significant.

### Nucleotide sequence accession numbers

The complete nucleotide sequences of 12 IndD4 isolates are available in DDBJ/EMBL/GenBank databases under accession numbers KF192830 -KF192841.

## Results

### Distribution of HBV genotypes and subgenotypes in Tripura

**Figure 1:**
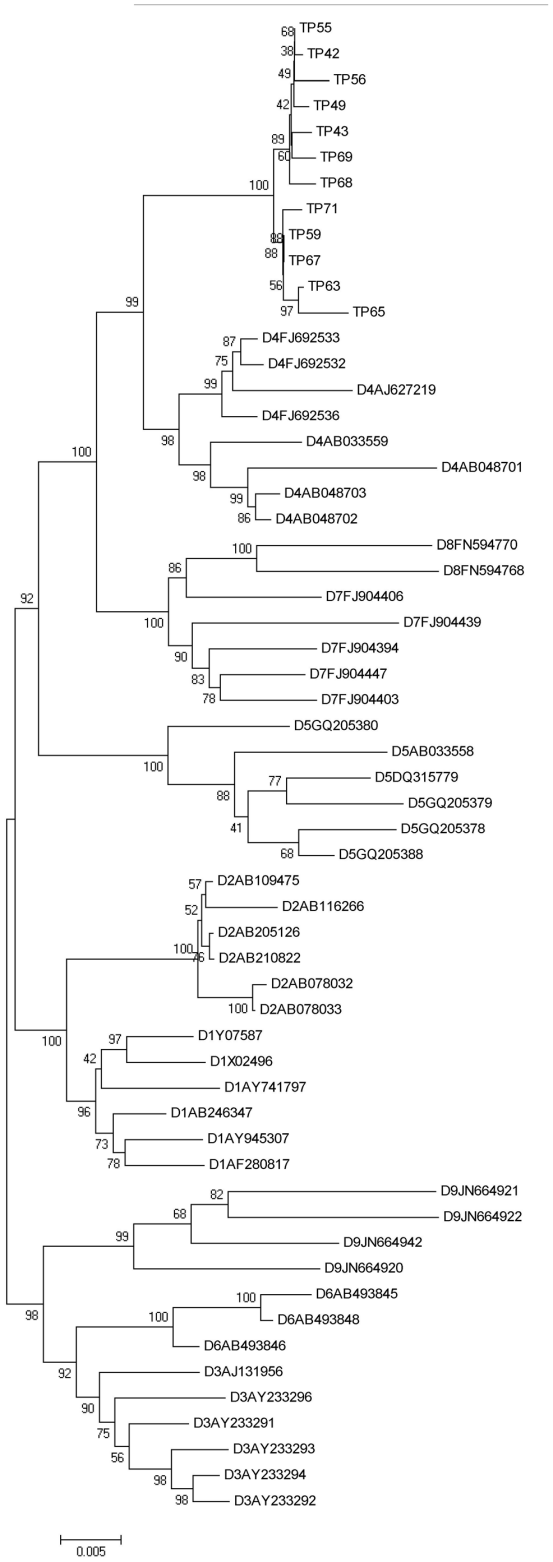
Phylogenetic tree of 12 Indian HBV strains belonging to subgenotype D4. The tree was formed on the basis of full-length HBV sequences. The reference sequences of different D-subgenotypes (D1-D9) retrieved from GenBank are indicated by their accession numbers. Indian HBV/D4 sequences of this present study are indicated by the respective isolate number beginning with “TP”.

**Figure 2:**
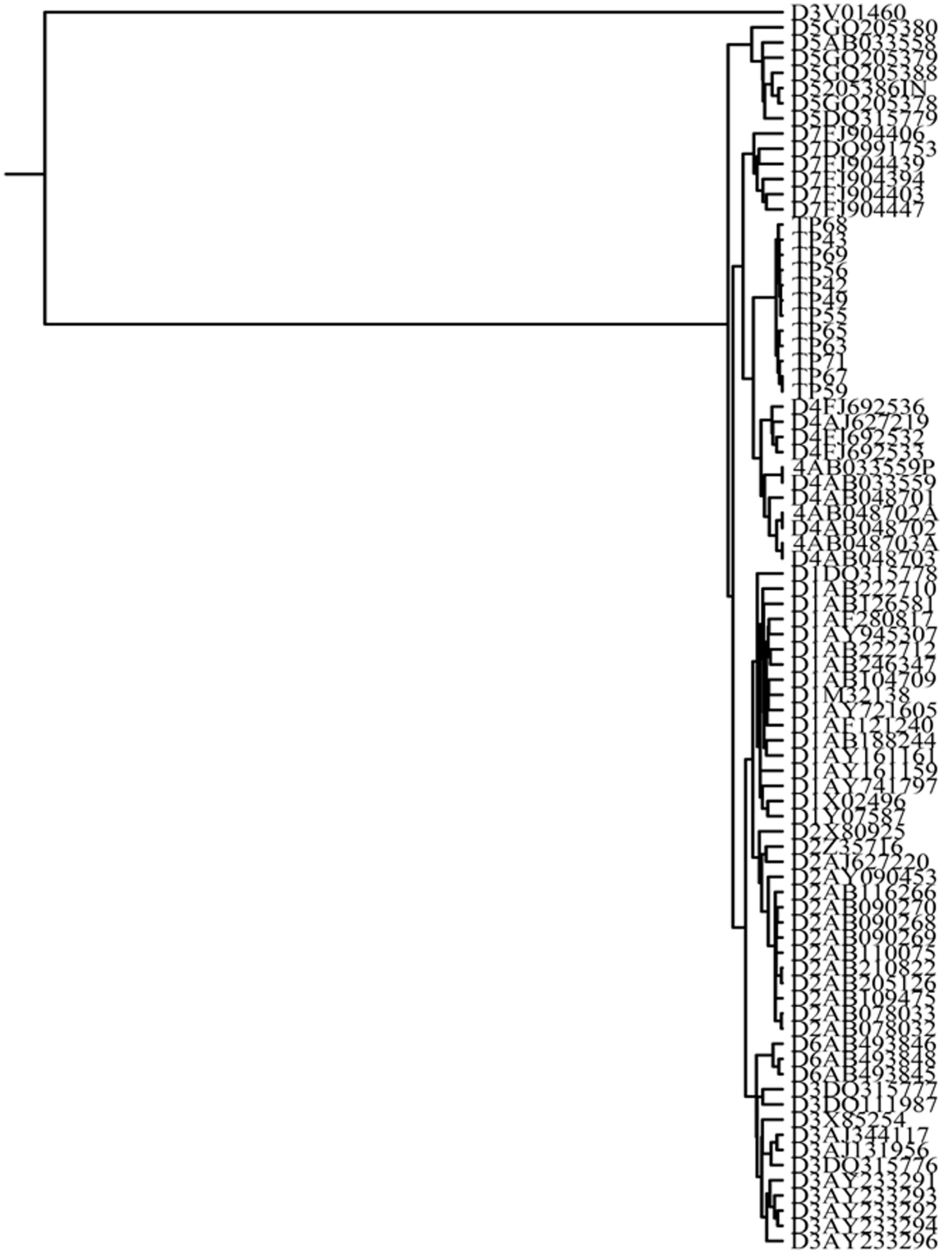
Estimation of divergence times of non-recombinant HBV subgenotypes D1–D7 including Indian HBV/D4 (IndD4). The tree was constructed by the DNAMLK program of the PHYLIP package, using indel-free HBV sequences of different subgenotypes of D, derived from GenBank, along with 12 IndD4 sequences. Reference sequences are indicated by their specific subgenotypes followed by accession numbers and the IndD4 sequences are indicated by respective isolate numbers beginning with “TP.”

The phylogenetic analysis of 76 PreS/S sequences of HBV from chronic carriers of Tripura revealed the presence of three distinct genotypes; D, C and A. Sixty eight HBV isolates (89.5%) belonged to genotype D, 5 (6.6%) were of genotype C and 3 (3.9%) clustered with genotype A (figure not shown). While all the HBV/A and HBV/C isolates belonged to subgenotype A1 and C1 respectively, genotype D strains were found to be most divergent and segregated into 5 distinct subsegenotypes. Thirty six of 68 HBV/D (52.94%) were found to cluster with subgenotype D3, 14 isolates (20.59%) were grouped in D2, 4 (5.88%) belonged to D1 and one (1.47%) to D5. Notably, the remaining 13 HBV/D sequences (19.1%) formed a separate clade within subgenotype D4 (Figure S1). HBV/D4 was not previously reported from any part of India and for a more detailed analysis of these strains, complete genome amplification and sequencing was successfully done in 12 D4-isolates. The phylogenetic tree constructed on basis of these full-length sequences once again placed the IndD4 strains in a separate clade within D4-subgenotype cluster, the branching being supported by high bootstrap value (Figure 1). The estimated intergroup nucleotide divergence (mean ± SD) between IndD4 and other global D4 sequences was found to be 2.7% while it was between 4% –5.2% with respect to other D-subgenotypes (Table 2). Moreover, none of the 12 HBV/D4 showed any evidence of recombination in their genome as determined by jpHMM method (data not shown). We examined the evolutionary relationships between IndD4 and other non-recombinant D-subgenotypes (D1–D7) and in our likelihood estimation of the tree (Figure 2), almost all representative strains were grouped according to their subgenotypes. The only exception was the sequence V01460, which was placed outside the D3 group and used as an outgroup to root the tree. The branching event showed that IndD4 was significantly distant and evolutionary more recent than D4 from other parts of the globe.

**Table 2:** Estimates of Evolutionary Divergence over Sequence Pairs between HBV/D subgenotypes (D1–D9) and Indian HBV/D4 (IndD4).

	D1	D2	D3	D4	D5	D6	D7	D8	D9	IndD4
**D1**										
**D2**	2.5±0.2									
**D3**	3.0±0.3	3.2±0.3								
**D4**	4.1±0.3	4.4±0.3	4.0±0.3							
**D5**	4.6±0.3	4.7±0.3	4.7±0.4	4.9±0.3						
**D6**	3.6±0.3	3.9±0.3	2.6±0.3	4.6±0.3	5.2±0.4					
**D7**	4.2±0.3	4.5±0.3	4.1±0.3	3.6±0.3	5.2±0.4	4.8±0.3				
**D8**	4.5±0.3	4.7±0.3	4.6±0.3	4.1±0.3	5.5±0.4	5.2±0.3	3.3±0.2			
**D9**	4.3±0.3	4.6±0.3	3.7±0.3	5.1±0.3	5.7±0.3	4.1±0.3	5.4±0.3	5.9±0.3		
**IndD4**	**4.0±0.3**	**4.3±0.3**	**4.0±0.4**	**2.7±0.2**	**4.8±0.4**	**4.4±0.4**	**3.7±0.3**	**4.2±0.3**	**5.2±0.3**	

Genetic distances of Indian HBV/D4 (IndD4) with other subgenotypes of D are indicated in bold and the genetic distance between IndD4 and reported D4 is enclosed within a box.

### Signature residues of HBV/IndD4 in different ORFs and regulatory regions

Prior to this study, a total of 15 full-length sequences of HBV/D4 were available in GenBank (Table S1). We compared the deduced amino acid sequences of all four overlapping open reading frames (ORFs), namely pre-S1/pre-S2/S, X, pre-C/C and P in all 12 IndD4 isolates with the existing D4 sequences as well as with other D-subgenotypes (D1–D9) to identify signature motifs of IndD4. IndD4 differed from the global HBV/D4 by 19 amino acid residues of which 12 residues were found in P ORF, one each in PreS1, PreS2 and PreC/C region and two each in S and X ORFs (Table 3).Of these 19 residues, 10 residues namely T^120^ in S region, L^11^ in X ORF, N^87^and E^90^ in the terminal protein (tp) region, F^30^ and A^62^ in spacer region (sp), N^128^, L^267^ and Q^333^ in reverse transcriptase (rt) domain and F^50^ in ribonuclease H (rh) region of ORF P are unique to IndD4, i.e., are not shared or exhibited by the other D-subgenotypes (Table 3) and can be considered as molecular signatures of IndD4.

**Table 3:** Conserved amino acid residues in four open reading frames of Indian HBV/D4 (IndD4) isolates compared with the previously reported residues at the same position in different subgenotypes of D (D1–D9).

Amino acid position	Amino acids in subgenotypes of HBV/D									
	D1	D2	D3	D4	D5	D6	D7	D8	D9	IndD4 [n = 12]
**PreS/S gene**										
PreS1-27*	T	**T**/S	T	T	T	T	T	H	T	S
PreS2-54	L	L	**L**/P	**L**/R	**P**/L	**Q**/L	L	S	**L**/P	Q
s120**	P	P	P	P	P	P	P	P	P	T
s129	**Q**/R	Q	Q	Q	**Q**/H	Q	**Q**/H	Q	Q	R
**PreC/C gene**										
c159	P	P	**P**/Q/T	P	P	**P**/Q	P	P	**T**/A/P	T
**X-Gene**										
x11	P	P	P	P	P	P	P	P	P	L
x12	A	**A**/S	A	A	A	A	**A**/T	A	A	T
**Polymerase Gene** ^a^										
tp87	D	D	D	D	D	D	D	D	D	N
tp90	K	K	K	K	**N**/Y	K	K	K	**K**/N	E
sp30*	H	**H**/L	H	H	H	Y	H	P	H	F
sp62	T	T	T	T	T	T	T	S	T	A
sp66	**P**/S	P	P	P	P	P	P	R	P	S
rt122	**F**/V	**F**/V	L	I	F	L	F	**F**/H	**L**/I/V	F
rt128**	T	T	T	T	T	T	T	T	T	N
rt267	Q	Q	Q	**H**/Y	H	Q	H	H	**Q**/H	L
rt333	K	K	K	K	K	K	K	K	K	Q
rh50	S	S	S	S	S	S	S	S	S	F
rh54	**I**/L	I	I	I	**I**/V	I	**I**/V	I	**I**/L	V
rh139	R	R	R	R	**R**/H	R	R	R	R	H

Unique residues of IndD4 are underlined. The major amino acids are indicated in **bold**. ^a^The conserved domains of the open reading frame of HBV polymerase are indicated as follows: tp, terminal protein domain; sp, spacer region; rt, reverse transcriptase domain; rh, RNase H region. *, ** the overlapping surface and polymerase mutations

It is noteworthy that T^120^ and R^129^ in S region, found in all IndD4 isolates (Table 3), are two reported HBV mutations with the potential to escape neutralization by vaccine induced antibody [7], [8] while N^128^ in rt domain of ORF P has been implicated as a compensatory or secondary mutation which could increase the viral fitness of HBV when exits in association with primary drug resistance mutation against Lamivudine [9].

The expression of HBV genes is controlled by four different promoters, two enhancers [EnhI [nt. 1074–1234] and EnhII [nt. 1636–1741], a negative regulatory element [NRE; nt. 1613–1636] and posttranscriptional regulatory elements [PRE] [∼nt 1151-1684]. Novel nucleotide polymorphisms have been perceived in all IndD4 isolates, two (A3021G and C3033T) in PreS2 promoter, one (A1126C) in EnhI and three (1310T, 1405T and 1407A) in PRE that were not seen in any of the D-subgenotypes (D1-D9) (Table 4).

**Table 4:** Signature nucleotide changes in the regulatory regions of Indian HBV/D4 (IndD4) isolates compared with the previously reported residues at the same position in different subgenotypes of D (D1-D9).

HBV Regulatory region	Nucleotide position	Nucleotide present in HBV/D subgenotypes									
		D1	D2	D3	D4	D5	D6	D7	D8	D9	IndD4 [n = 12]
Enhancer I/X promoter [nt 1074–1234]	1126	A	A	A	A	A	A	A	A	A	C
S2 Promoter [∼nt 2971–3127]	3021	A	A	A	A	A	A	A	A	A	G
	3033	C	C	C	C	C	C	C	C	C	T
NRE [∼nt 1613–1636]	1633	**A**/G	A	**A**/G	**A**/G	A	A	**A**/G	A	A	G
	1310	C	C	C	C	C	C	C	C	C	T
	1321	A/C	A	A	A	**A**/G	A	**A**/G	A	**A**/C	G
	1368	**G**/A	**A**/G	**G**/A	G	**G**/A	A	G	G	**G**/A/C	C
PRE [∼nt 1151–1684]	1405	C	C	C	C	C	C	C	C	C	T
	1407	G	**G**/T	G	G	G	G	**G**/T	G	G	A
	1577	G	G	G	G	**G**/A	G	G	G	G	A
	1633	**A**/G	A	**A**/G	**A**/G	A	A	**A**/G	A	A	G

Unique residues of IndD4 are underlined. The major nucleotide substitutions are indicated in **bold**.

### Biochemical and virological characteristics of infection with IndD4 in comparison with other D-subgenotypes

We compared the clinical and virological characteristics of subjects infected with HBV subgenotypes D1-IndD4, while excluding the single patient carrying D5. A predominance of chronic HBeAg-negative infection was noted in Tripura where all subjects harboring IndD4 or D3 and 75% with D1 were negative for HBeAg (Table 5). Majority of patients with IndD4 had ALT <40 IU/L, similar to those with HBV/D2, while AST level as well as HBV viral load was significantly low in IndD4 as compared to other 3 subgenotypes (D1–D3) (p<0.05) (Table 5). Mutations in BCP and PC region of HBV that can decrease or prevent the synthesis of HBeAg were analyzed in different D-subgenotypes of HBeAg-negative patients. The double mutation (A1762T and G1764A) in BCP was undetected in IndD4 although it was found at a frequency of 33.33% in D1, 8.33% in D2 and 9.09% in D3 isolates. The stop-codon mutation G1896A in PC was noted in 8.33% of each of IndD4 and D2 and in 9.09% of HBV/D3 while it was absent in D1.

**Table 5:** Demographic, serological and virological characteristics of HBV/D (D1-IndD4) samples.

HBV/D subgenotype	D1 [n = 4]	D2 [n = 14]	D3 [n = 36]	IndD4 [n = 13]	p-value [D1, D2, D3 and IndD4]	p-value [D1, D2, D3 group vs IndD4]
**Age [years], mean±SD**	41.00±12.70	32.77±13.31	37.86±12.29	30.31±9.49		
**ALT [IU/l], Median, Range**	59.75 [21.00 – 72.50]	39.75 [30.00– 96.10]	47.05 [15.60– 145.90]	38.00 [18.00– 62.00]	0.3852^$^	0.1593#
**AST [IU/l], Median, Range**	38.20 [34.00 – 46.20]	49.00 [32.10– 72.00]	49.85 [17.70– 110.0]	32.00 [10.00– 52.00]	0.0069*^$^	0.0005*#
**HBV DNA [IU/ml], Median, Range**	2.41×10^2^ [14.21 – 1.7×10^8^]	9.15×10^6^ [3.4×10^5^ – 1.7×10^8^]	3.09×10^3^ [79 – 6.9×10^4^]	8.6×10^1^ [20– 7.99×10^2^]	0.0002*^$^	0.0222* #
**HBeAg negativity [%]**	75	57.14	100	100		

SD, standard deviation; ALT, alanine aminotransferase; AST, aspartate aminotransferase; IU, international unit; #Mann-Whitney test p-value; $One-way ANOVA p value; *p-value <0.05 was considered significant.

## Discussion

In the present study we investigated the prevalence of HBV genotypes/subgenotypes among chronically HBV infected patients in the N-E Indian state of Tripura that is geographically remote from the heartland of India but has rich ethnic diversity and cultural heritage. HBV/D was predominant in Tripura, similar to that observed in most parts of India [3], although genotypes C and A were also detected, albeit at much lower frequencies. However, few prior studies from other N-E states indicated that HBV/A was the major genotype in Arunachal Pradesh followed by genotypes C and D [10]; HBV/D was most common in Assam followed by genotypes A and C [11] while in Manipur, the occult HBV from HIV-positive injection drug users belonged mostly to genotype C [12], thereby reflecting a distinct distribution of viral genotypes in different N-E states.

Phylogenetic analysis of HBV/D isolates of Tripura revealed the presence of five D-subgenotypes (D1-D5), the most striking aspect being the detection of subgenotype D4 that is rare in the world and till date had not been reported from any part of India. Globally, HBV/D4 has been found in the African nation of Rwanda [13] and sporadically in Somalia [13], Kenya [14], Ghana [15] and Morocco [16]. It is also found in the Caribbean islands of Haiti [13] and Martinique [17] where its spread was believed to be linked to slave transport from Africa to Caribbean. This subgenotype has also been encountered in ethnic Micronesian community of the Solomon Islands [18], in Aborigines from Australia, in a blood donor from Papua, New Guinea [18], [19] as well as in Arctic-native Dene population of Canada [20]. HBV/D4 has been reported from the Brazilian state of Rondônia whose inhabitants are mainly descendants from European colonizers, African slaves, and Native Americans [21]. In this study we observed that ∼19% of HBV/D sequences from Tripura belonged to D4. Both partial and complete genome analyses showed that these strains formed a distinct clade within D4, with a high bootstrap value, segregating from both African and Australian D4 isolates.

Molecular epidemiology of HBV suggests that it followed modern human since their dispersal from Africa and the genesis of global genetic tapestry of HBV and the emergence of different genotype/subgenotype resulted from multiple founder events that occurred during their long march with their host across the continents [22]. Given that a sizeable percentage of HBV/D4 was found in individuals of African origin, it is tempting to speculate that D4 might have originated in Africa and had followed the early wave of human migration via a southern route around the coasts of Indian Ocean to South-East Asia and ultimately to Melanesia and Australia [23], [24]. The spread of D4 among the Native Americans (including Na-Den'e Indians) might be linked with later migratory events that occurred subsequent to the colonization of East Asia and the northward diffusion of Asian migrants as far as Siberia and their arrival into American continent from the Beringian landmass [25]. The introduction of HBV/D4 in India might be more recent and given its sole presence in N-E India, it could be envisaged that its advent might have been associated with the settlement of proto-Tibeto-Burman people, who are exclusive to this region and had migrated from East Asia [26]. Our analysis of D4 sequences from different geographical regions and calibrating a molecular clock based on observed nucleotide changes suggested that IndD4 diverged more recently than African and Australian isolates, giving credence to our hypothesis of their relatively recent arrival to India. The narrow strip of land that connects the N-E Indian region to the remaining part of India has served has a bottleneck to the dispersal of Tibeto-Burman immigrants who thus remained land-locked in this region and concurrently HBV/D4 might have been circulating and evolving in isolation in this region for many years unnoticed. Tripura is home to a plethora of ethnic groups, most of whom derived their ancestry from Tibeto-Burman family and future studies of HBV genotype/subgenotype in these primitive communities can shed more light into the genetic geography of the virus as well as the as the history and demography of its host and viral transmission between communities.

The multiple episodes of human migration had also been associated with their significant genetic differentiation and by extrapolation, seem to have influenced the evolutionary trajectory of HBV that is reflected in the emergence of unique signature residues across its four ORFs and regulatory regions. By comparing the amino acids of IndD4 with the available global D4 as well as with other D-subgenotypes, we were able to identify 10 signature residues that were exclusive to IndD4. The most clinically relevant residues found in all IndD4 were T^120^ and R^129^, present within the major hydrophilic region (aa 99-169) of S ORF. It has been reported that T^120^ and R^129^ can cause reduced binding affinity to anti-HBs or vaccine-induced antibodies [7],[8], which may contribute to the establishment of persistent infection in both unvaccinated and vaccinated individuals. Moreover, both these residues, particularly R^129^, significantly reduced the extracellular level of S proteins and also impair the secretion of subviral particles and virions *in vitro* and *in vivo* [27]. This may result in HBsAg detection failure, misdiagnosis of hepatitis B among HBV/D4 cases and transmission of HBV from ‘occult’ infected blood donors. Moreover it also provides a plausible explanation to the low viral titers observed in individuals with IndD4. Reduced virion secretion may also cause the intrahepatic retention of virions and S proteins, which can induce endoplasmic reticulum stress and promote inflammation [28] and thus a long term carriage of IndD4 may lead to more severe liver disease. The presence of T^120^ in S ORF also creates the rtT128N exchange in overlapping P ORF. It has been reported that rtN^128^ substitution can restore the replication fitness of lamivudine-resistant HBV mutants carrying changes in YMDD motif in presence of the drug [9], thereby limiting the long-term efficacy of nucleoside analogue therapy for IndD4-bearing patients and supporting the need of their close surveillance and treatment adjustments. All IndD4 also harbored novel nucleotide substitutions in PreS2 promoter, EnhI and PRE but their implications on HBV transcription and replication and their impact on viral pathogenicity warrants further investigation.

Since the patients with IndD4 maintained a significantly low viral load as compared to those with other D-subgenotypes, it appears that IndD4 may have an inherently low replicative capacity. While a rapidly replicating virus through generation of a larger antigen pool could be easily recognized by host immune system, a lowly replicating virus, such as IndD4, would evoke a weaker immune response and thereby have an increased chance of persistence that in turn may result in progressive liver disease.

The absolute predominance of HBeAg-negative infection and the near complete absence of BCP/PC mutations characterize the IndD4 infected patients. It has been shown that the genetic diversity of HBV is highest during periods of high viral replication and a mounting immune response, while the mutation rate appears to be low, when the host immune response is relatively quiescent [29]. Most IndD4 carrying HBeAg-negative subjects in the present study had biochemically inactive infection and it is possible that the relatively quiescent and inactive virological status and stable immunological status negatively influenced the emergence and evolution of PC/BCP mutants as the predominant species over the wild-type sequences.

Taken together, the relative isolation of Tripura and its ethnic constitution served as a protection against the spread of HBV/IndD4 and may explain its absence in other parts of the country. The study also reiterates the fact the HBV can serve as an ideal surrogate marker to study human population movements and ancestry and can help to unravel many strongly debated topics in human history. Although IndD4 may appear rather innocuous to its carriers owing to low viral load and normal levels of ALT/AST but the presence of unique genetic signatures that favor immune/vaccine escape, drug resistance and long-term persistence in host can make it an emerging clinical challenge with the potential of undermining the success of vaccination programs or anti-viral therapy.

## Supporting Information

Figure S1 **Phylogenetic tree showing the subgenotypic distribution of 68 HBV strains belonging to genotype D from Tripura.** The tree was formed on the basis of PreS/S sequences. The reference sequences of different subgenotypes of D, namely, D1 to D9, retrieved from GenBank are indicated by their accession numbers while HBV/D sequences determined in the present study are indicated by their respective isolate numbers beginning with “TP”.
(JPG)

Table S1 **GenBank Accession numbers and country of origin of 15 full length sequences of HBV of subgenotype D4 available prior to the present study.**
(DOC)
