# Supplementary figures and images for: A Rare HBV Subgenotype D4 with Unique Genomic Signatures Identified in North-Eastern India –An Emerging Clinical Challenge?
Source: PLoS One. 2014 Oct 8;9(10):e109425. doi: 10.1371/journal.pone.0109425 (PMC4190083; doi:10.1371/journal.pone.0109425)

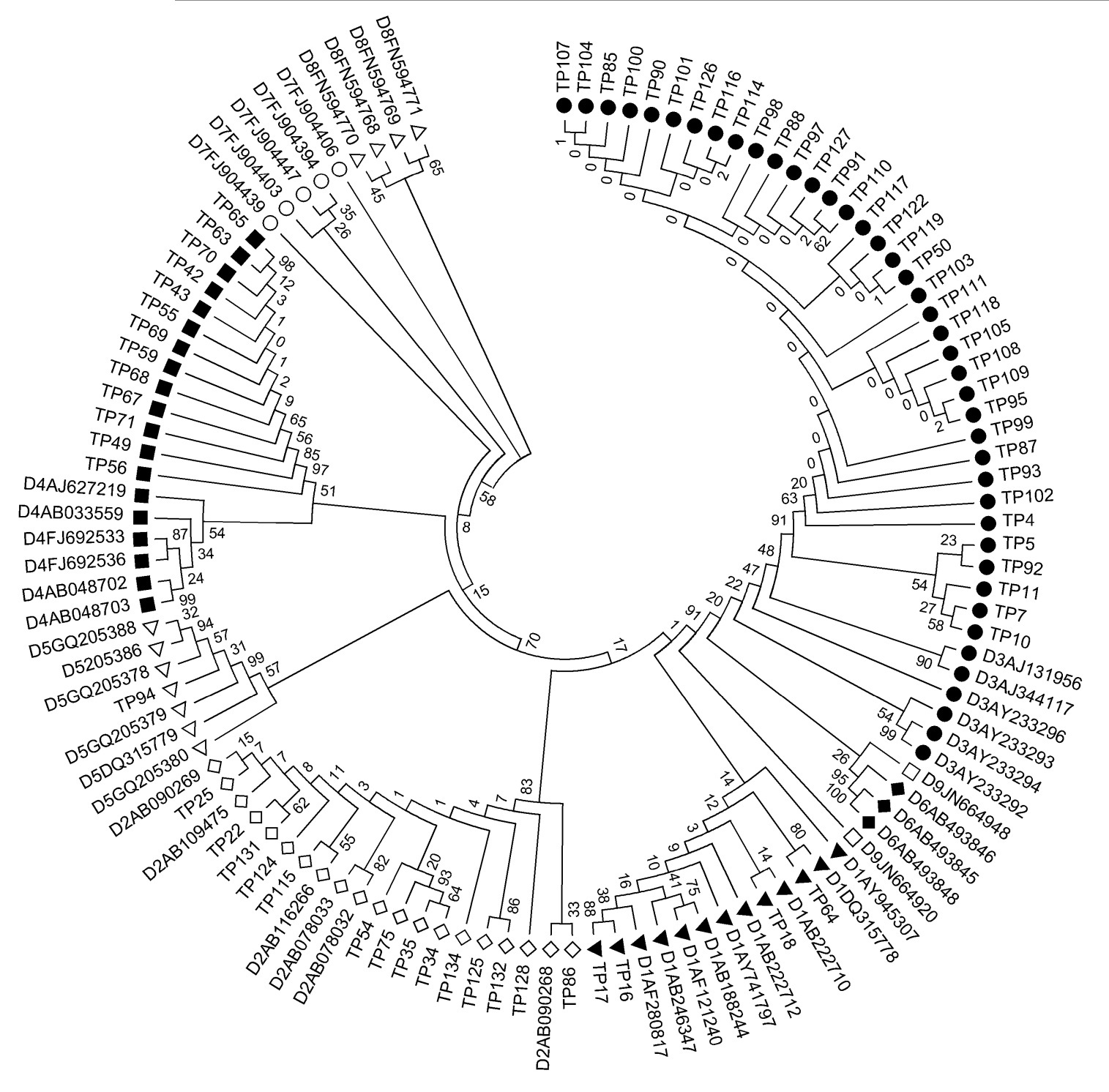

Supplement: Figure S1 — Phylogenetic tree showing the subgenotypic distribution of 68 HBV strains belonging to genotype D from Tripura. The tree was formed on the basis of PreS/S sequences. The reference sequences of different subgenotypes of D, namely, D1 to D9, retrieved from GenBank are indicated by their accession numbers while HBV/D sequences determined in the present study are indicated by their respective isolate numbers beginning with “TP”. (JPG) [file pone.0109425.s001.jpg]
