# Supplementary material for: A Rare HBV Subgenotype D4 with Unique Genomic Signatures Identified in North-Eastern India –An Emerging Clinical Challenge?
Source: PLoS One. 2014 Oct 8;9(10):e109425. doi: 10.1371/journal.pone.0109425 (PMC4190083; doi:10.1371/journal.pone.0109425)
Supplement: Table S1 — GenBank Accession numbers and country of origin of 15 full length sequences of HBV of subgenotype D4 available prior to the present study. (DOC) [file pone.0109425.s002.doc]

**Supplementary table 1:** GenBank Accession numbers and country of origin of 15 full length sequences of HBV of subgenotype D4 available prior to the present study

| **GenBank Accession No. of HBV/D4 sequences** | **Country of origin** |
| --- | --- |
| FJ692532 | Haiti |
| FJ692533 | Haiti |
| FJ692536 | Haiti |
| AJ627219 | Spain |
| AB048701 | Australia |
| AB048702 | Australia |
| AB048703 | Australia |
| AB033559 | Papua |
| HE974372 | Martinique |
| HE974373 | Martinique |
| HE974378 | Martinique |
| HE974382 | Martinique |
| GQ922003 | Canada |
| GQ922004 | Canada |
| GQ922005 | Canada |
